# Does dynamic navigation assisted student training improve the accuracy of dental implant placement by postgraduate dental students: an in vitro study

**DOI:** 10.1186/s12903-024-04324-4

**Published:** 2024-05-23

**Authors:** Qi Yan, Xinyu Wu, Junyu Shi, Bin Shi

**Affiliations:** 1 Department of Oral Implantology, The State Key Laboratory Breeding Base of Basic Sciences of Stomatology and Key Laboratory of Oral Biomedicine, Ministry of Education (Hubei-MOST KLOS & KLOBM), School and Hospital of Stomatology, Wuhan University, Wuhan, China; 2 Department of Oral and Maxillo-Facial Implantology, Shanghai Ninth People’s Hospital, Shanghai Jiao Tong University School of Medicine, College of Stomatology, Shanghai Jiao Tong University, National Center for Stomatology, National Clinical Research Center for Oral Diseases, Shanghai Key Laboratory of Stomatology, Shanghai, China

**Keywords:** Dental implants, Dynamic guided navigation, Education

## Abstract

**Objectives:**

To assess the accuracy of implant placement in models and satisfaction in dynamic navigation assisted postgraduate dental students training.

**Methods:**

Postgraduate dental students who had at least one year of dental clinical practice with no experience in dental implant surgeries were included. Students were instructed to make treatment plans in the dynamic navigation system. Each student placed two maxillary right incisors, using freehand approach at first and then under dynamic navigation. The implant position was compared with treatment plan. Factors influencing the accuracy of implants placed under dynamic navigation were analyzed. Student acceptance towards the training and use of dynamic navigation was recorded using a questionnaire.

**Results:**

A total of 21 students placed 42 implants. For freehand implant placement, the median entry point deviation, apex point deviation, and implant axis deviation was 3.79 mm, 4.32 mm, and 10.08°. For dynamic guided implant placement, the median entry point deviation, apex point deviation, and implant axis deviation was 1.29 mm, 1.25 mm, and 4.89° (*p* < 0.001). The accuracy of dynamic guided implant was not influenced by student gender or familiarity with computer games. All students were satisfied with the training.

**Conclusions:**

Dynamic navigation system assisted students in improving the accuracy of implant placement and was well accepted by students.

## Introduction

Tooth loss is prevalent in the world, having a negative impact on the well-being of hundreds of millions of people [1, 2]. Dental implants have become a common method of rehabilitating missing teeth. An increasing number of well-trained clinicians performing implant surgeries is required to meet the needs of patients.

Training in surgical procedures is a major part of education in implant dentistry. Students should be trained to have competence in designing a prosthesis-guided implant position and being familiar with surgical procedures. However, current education in implant dentistry mainly focuses on theory [3]. According to previous surveys, only 5% of students operated on patients in clinical practice in Europe [4] and students had an average of 0.61 surgical cases in America [5]. Lack of surgical skill training in implant dentistry could be attributed to a lack of staff availability/competence and suitable patients [6]. In addition, it is risky and not ethical for patients if an untrained clinician performs the surgery.

Dynamic navigation has been widely used as a method of student training in oral and maxillofacial surgery [7] and root canal treatment [8]. In implant dentistry, dynamic navigation is beneficial to improve the accuracy of implant placement [9–12] and provides an alternative to training in surgical procedures. Through dynamic navigation, students can design the three-dimensional implant position in software, visualize the implant site on a computer screen, receive real-time feedback and make adjustments, and compare the final implant position with the presurgical design. Previous studies have reported that dynamic guided navigation well-assisted students in improving the accuracy of implant placement and helped students improve their familiarity of surgical skills and confidence [13, 14]. In addition, a recent study reported that dynamic navigation improved the accuracy of implant placement by dental students [15]. However, it is unclear whether dynamic navigation is well accepted by students with totally no surgical experience in implant dentistry. Furthermore, to improve the quality of student training, factors influencing the accuracy of dynamic guided navigation surgeries performed by students should be explored.

Thus, in the present study, postgraduate dental students with no surgical experience were instructed to place two implants, using a freehand approach and under dynamic navigation. The accuracy of implant position using the two approaches was compared to evaluate the benefit of dynamic navigation in surgery training. In addition, factors influencing the accuracy of dynamic guided navigation surgery were investigated.

## Materials and methods

The study protocol was approved by the institutional review board of the Hospital of Stomatology, Wuhan University (No. 2022[B18]). Postgraduate students who had at least one year of dental clinical practice, in the Hospital of Stomatology Wuhan University, with no surgical experience in dental implant placement, and who had not participated in previous courses, were included.

Students had received courses in implant dentistry before participating in this training course. The present course emphasized implant placement surgery and digital plans. The course included a theoretical part and a practical part. The theoretical course included the biological basis for peri-implant tissue (3 h), patient examination (2 h), treatment plan protocols (3 h), digital treatment plan (3 h), as well as surgical procedures for osteotomy preparation and implant insertion (2 h). The practical course included training on digital planning in software (1 h), freehand, and dynamic navigation assisted implant placement in models (1 h). During the practical training, each student was assigned to place two implants (4.1*10 mm, Straumann, Bone level tapered implants, Switzerland) to replace the maxillary right incisor in models. In the beginning, students made treatment plans in software (Dental Implant Navigation System v2.5.1, Digital-health Care Co.Ltd., Suzhou, China) with the consultation of an experienced surgeon. Then, students were instructed to place one implant using a conventional freehand approach and place a second implant under dynamic navigation (Digital-health Care Co.Ltd., Suzhou, China).

Before the training course, 3D-printed photosensitive resin maxillary models with missing right incisors were prepared (Digital-health Care Co.Ltd., Suzhou, China). The same model was used for all students. Radiopaque markers were placed on the buccal and palatal of the models, for 3D orientation of the models and subsequent superimposition. CBCT scan (NewTomVGi, Italy) of the model was taken and the DICOM file was imported into the treatment plan software. To prepare for the training, the models were stabilized onto simulation heads (Type 2 simulation head, NISSIN, Japan). The models were registered to be spatially matched to the virtual representation on screen by a single trained engineer. During osteotomy preparation, the drills were calibrated, to provide the system with information on drill position and length. The accuracy of drill position and angulation was monitored and real-time feedback was given on screen.

**Fig. 1:**
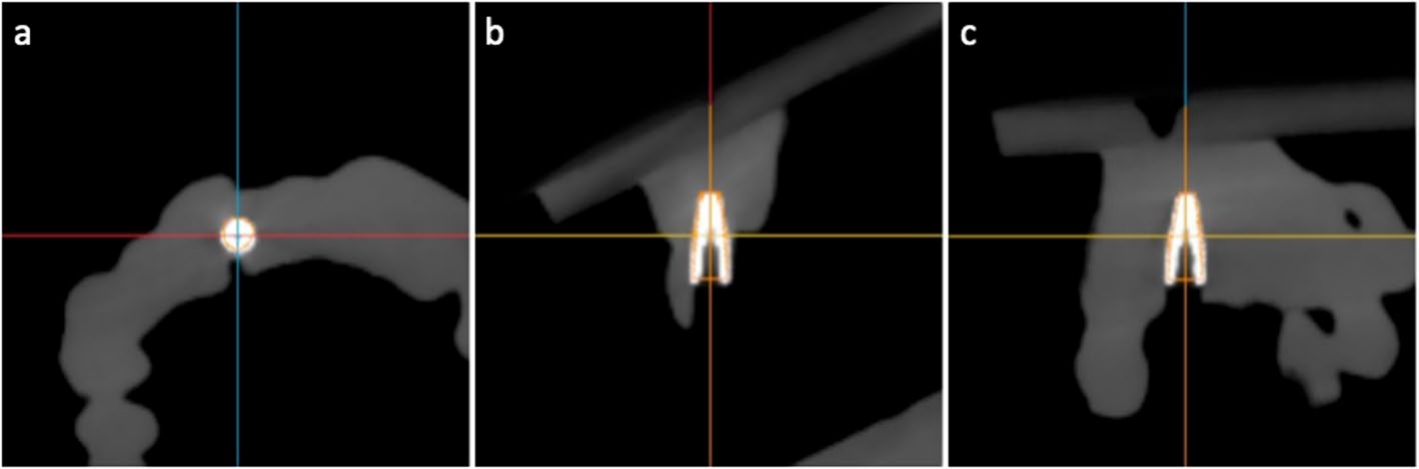
Measurement of the accuracy of implant placement. **a** implant coronal plane; **b** implant sagittal plane; **c** implant axial plane

After implant placement, CBCT scans of all models were taken and imported into an accuracy validation software (Computer Assisted Dental Implant System v2.5.1, Digital-health Care Co.Ltd., Suzhou, China). Pre- and post-surgery CBCT was superimposed using three to five anatomy markers. The coordination of the plan and actual implants were automatically detected and manually checked. The positional deviation of the plan and actual implants was calculated, including entry point deviation, apex point deviation, and implant axis deviation (Fig. 1). The measurement was performed by one calibrated researcher (with an ICC of 0.97).

After the course, students were asked to finish a questionnaire, including students’ attitudes towards the content and atmosphere of the courses, application of dynamic navigation, interest in future study, as well as overall satisfaction [14, 16–19]. The degree of satisfaction was ranked using a Likert scale, including “strongly satisfied,” “satisfied,” “neither satisfied nor unsatisfied,” “unsatisfied,” and “strongly unsatisfied” [20].

SPSS 25.0 (IBM Corp, United States) was used for data analysis. Shapiro–Wilk test indicated that the deviation of implant position did not follow the normal distribution. The median and interquartile range were used for descriptive data. Wilcoxon matched-pairs signed rank sum test was used to compare the accuracy of implant placement using a freehand approach and under dynamic navigation. To explore factors associated with the accuracy of implant placement under dynamic navigation, the Mann–Whitney U test was used. Potential factors included student gender and whether they were familiar with computer games (familiar vs, not familiar). *P* < *0.05* was considered statistically significant.

## Results

A total of 21 postgraduate students were enrolled, of which 13 (61.9%) were females, six (28.6%) majored in implant dentistry, 17 (81.0%) spent at least two years in dental clinical practice, and nine (42.9%) reported they were familiar with computer games.

The accuracy of implant placement is listed in Table 1. Compared with treatment plan, the median (interquartile range) entry point deviation, apex point deviation, and implant axis deviation of freehand implant placement were 3.79 (2.80, 4.60) mm, 4.32 (3.23, 5.14) mm, and 10.08 (7.35, 14.99) °. The median (interquartile range) entry point deviation, apex point deviation, and implant axis deviation of dynamic guided implant placement were 1.29 (1.08, 2.00) mm, 1.25 (1.02, 1.65) mm, and 4.89 (2.27, 6.80) °. The deviation of dynamic navigation was significantly less than the freehand approach. No factor was found significantly associated with the accuracy of implant placement under dynamic navigation or freehand surgery (Table 2).

**Table 1:** Accuracy of implant placement using freehand approach and dynamic navigation

	**Entry point**^**a**^	**Apex point**^**a**^	**Angulation**^**a**^
Free hand	3.79 (2.80, 4.60)	4.32 (3.23, 5.14)	10.08 (7.35, 14.99)
Dynamic navigation	1.29 (1.08, 2.00)	1.25 (1.02, 1.65)	4.89 (2.27, 6.80)
*p value*^‡^	< *0.001*	< *0.001*	< *0.001*

^‡^Wilcoxon matched-pairs signed rank test

^a^The data was described as median (interquartile range)

**Table 2:** Factors associated with the accuracy of dynamic navigation and freehand implant placement

	**Dynamic navigation**			**Freehand**		
	**Entry point**^**a**^	**Apex point**^**a**^	**Angulation**^**a**^	**Entry point**^**a**^	**Apex point**^**a**^	**Angulation**^**a**^
Gender						
Male	1.51 (1.24, 3.10)	1.81 (0.90, 2.93)	6.01 (2.41, 6.99)	4.12 (3.27, 4.72)	4.46 (3.38, 6.44)	15.45 (9.07, 18.40)
Female	1.20 (1.06, 1.48)	1.26 (1.08, 1.54)	4.14 (1.86, 5.74)	3.70 (2.46, 4.51)	4.25 (2.98, 5.06)	9.04 (5.72, 11.82)
* p value*	*0.149*	*0.986*	*0.585*	*0.5846*	*0.4880*	*0.0667*
Computer game						
Not familiar	1.20 (1.05, 2.71)	1.33 (1.13, 2.81)	4.14 (2.14, 7.31)	4.05 (3.04, 4.80)	4.44 (3.85, 5.12)	10.53 (8.07, 14.49)
Familiar	1.35 (1.20, 1.84)	1.16 (0.79, 1.34)	4.92 (1.75, 6.31)	3.52 (2.62, 4.60)	4.32 (3.06, 5.79)	9.07 (6.28, 16.34)
* p value*^‡^	*0.602*	*0.153*	*0.862*	*0.4221*	*0.8621*	*0.8078*

^‡^Mann–Whitney U test

^a^The data was described as median (interquartile range)

Student acceptance towards the training course is shown in Table 3. For all items, no student reported “unsatisfied” or “strongly unsatisfied”. All students were satisfied with the training interest and perspective. A majority of students showed positive attitudes towards the content and atmosphere of the training. More than 90% of the students were willing to participate in future training. Overall, all students were satisfied with the dynamic guided implant placement training.

**Table 3:** Student acceptance towards the training using dynamic navigation system

Questionnaires	Strongly satisfied n (%)	Satisfied n (%)	Neither satisfied nor unsatisfied n (%)
Attitudes towards course content			
Course focus	12 (57.1)	8 (38.1)	1 (4.8)
Course interest	16 (76.2)	5 (23.8)	0 (0)
Course richness	10 (47.6)	9 (42.9)	2 (9.5)
Course usefulness	14 (66.7)	4 (19.0)	3 (14.3)
Course prospective	16 (76.2)	5 (23.8)	0 (0)
Acquisition of knowledge	5 (23.8)	9 (42.9)	7 (33.3)
Combine theory with practice	13 (61.9)	4 (19.0)	3 (14.3)
Course content satisfaction	14 (66.7)	6 (28.6)	1 (4.8)
Attitudes towards course atmosphere			
The activity of class atmosphere	13 (61.9)	7 (33.3)	1 (4.8)
Satisfaction with the use of dynamic navigation	13 (61.9)	7 (33.3)	1 (4.8)
Interaction between teachers and students	14 (66.7)	6 (28.6)	1 (4.8)
Attitudes towards dynamic navigation			
Improvement of clinical skill	11 (52.4)	8 (38.1)	2 (9.5)
Improve the ability of pre-clinical practice	11 (52.4)	8 (38.1)	2 (9.5)
Improve the operational process of practical task	12 (57.1)	7 (33.3)	2 (9.5)
Improve the operational outcome of practical task	11 (52.4)	9 (42.9)	1 (4.8)
It makes easy for me to understand the essentials of the practical task	11 (52.4)	8 (38.1)	2 (9.5)
Attitudes towards future study			
Improvement of learning motivation	12 (57.1)	8 (38.1)	1 (4.8)
Take part in future course	18 (85.7)	2 (9.5)	1 (4.8)
Overall satisfaction	14 (66.7)	7 (33.3)	0 (0)

A post hoc sample size calculation was performed to verify the results. The superiority by margin tests for the difference between two means in PASS (15.0) were used, with α = 0.01, and power = 0.8. Accuracy at entry point, apex point, and angular deviation were used. Six, 6, and 13 implants in each group were needed to detect differences regarding accuracy at the entry point, apex point, and angular deviation.

## Discussion

The present study compared the accuracy of dental implant placement by postgraduate students using a freehand approach or dynamic navigation. Dynamic navigation significantly improved the accuracy of dental implants placed by students with no surgery experience, and was well accepted by students. The results indicated that dynamic navigation could be successfully used in surgery training of implant placement for dental students, regardless of gender or familiarity with computer games.

In the present study, the accuracy of implant placement by students improved significantly after using dynamic navigation. A previous model-based study [21] reported that freehand implant placement yielded a deviation of 1.44 mm at entry points, 2.00 mm at apex points, and 9.66° for implant axis, which was smaller compared with the deviation produced by freehand implant placement in this study. It could be explained that in this study implants were placed by students with no surgery experience. However, another study [22] reported that using dynamic guided navigation surgeries in models, the mean entry point deviation, apex point deviation, and implant axis deviation were 0.91 mm, 1.21 mm, and 2.78°, respectively, which was similar to the results of the present study. It was indicated that dynamic guided navigation could help students place implants in models.

The present study did not find factors associated with the accuracy of implants placed by students using dynamic navigation. A previous study reported that computer games might be beneficial for interactive virtual guidance [23]. Because they shared the characteristics that a player/dentist looked at the screen, received real-time feedback, and operated on hand. Another study reported a slight learning advantage of dynamic guided implant placement for male students [13]. They explained that males showed learning advantages because they were more familiar with video games. However, the present study did not find significant different accuracy between students familiar or not familiar with computer games. It was indicated that dynamic navigation could improve accuracy of implant placement, regardless of the gender or familiarity with computer games.

The training using dynamic guided surgery was well accepted by students, which is similar with previous studies reporting the learning curve of dynamic navigation [24, 25]. On the one hand, the digital workflow of dental implant-supported rehabilitation is developing rapidly. It is important for the surgeons to master dynamic guided navigation surgery, treat patients with compromised clinical conditions, and broaden the indications of dental implants. On the other hand, dynamic navigation benefits students in placing dental implants accurately, which might be preferred by dental students. However, the clinical conditions are usually more complex and the accuracy of implants is only part of the success of implant-supported rehabilitation. Thus, although the dynamic navigation system could help improve the accuracy of dental implants placed by students, it could not be a substitute for clinical practices.

Results from the present study indicated that dynamic navigation was beneficial to student training in dental implant placement. Practically, the advantages of dynamic navigation in student training include visualization of implant placement procedure, better accuracy, and a smoother learning curve. However, the economic cost of student training using dynamic navigation is relatively high. Dynamic navigation is not suitable for all clinical cases. The accuracy of dynamic navigation is dependent on the accuracy of pre-surgery preparation, including registration, calibration, and the inner algorithm of the system. In the future, the development of dynamic navigation and promotion of potential augmented reality can be beneficial to further improve student training in dental implant placement.

One limitation of the present study was that all implants were placed in the maxillary right incisor and the influence of implant site on accuracy was not explored. Maxillary right incisor was chosen because implant osteotomy could be directly visualized and the complexity of surgery was reduced for students. However, the influence of implant sites and extensive edentulous ridge on the accuracy of dynamic guided implant placement has been reported in previous clinical studies or model-based studies [22, 26]. Secondly, a comparison of the accuracy of dynamic guided implant placement between experienced and inexperienced dentists was not performed, which was beyond the scope of this study and has been analyzed in previous research [27, 28]. Thirdly, students performed first freehand implant placement and then navigation assisted implant placement. The order was not randomized, which might cause bias. However, the dynamic navigation assisted implant placement required clinicians to have some experience in implant placement. The postgraduate students had no experience with surgical interventions in implant dentistry. They were asked to first perform freehand implant placement to help the students get familiar with the models and drilling procedures.

In the future, research with larger sample sizes or randomized trials is recommended, to compare the dynamic guided navigation training with conventional training. Dynamic navigation equipment with different subject generations can be investigated and explored to improve the accuracy and promote the usage of dynamic navigation in student training. In addition, it could be further explored whether dynamic guided navigation could be applied in the training of other surgical techniques, such as sinus floor elevation, immediate implant placement, or alveolar ridge augmentation.

## Conclusions

The dynamic guided navigation system was successfully applied in training implant placement among dental students. The accuracy of implant placement by dental students could be improved under dynamic guided navigation, regardless of student gender or familiarity with computer games. The application of dynamic guided navigation in surgery training is well accepted by students and could be promoted in dental student education.

## Data Availability

The datasets used and/or analysed during the current study are available from the corresponding author on reasonable request.
